# Annotations

**Published:** 1896-04-04

**Authors:** 


### Annotations.

### The Clinical Society.

Several good papers were read at the last meeting of the Clinical Society, but undoubtedly the interest of the evening centred in that of Mr. Mayo Robson, in which he gave a careful review of the comparative merits of the various methods which are in common use for securing union of the divided edges of hollow viscera. He arranged these methods in three main groups. (a) Simple suturing, without any mechanical aid; (b) the production of adhesion by some such apparatus as Murphy's button, by which a line of pressure-necrosis is produced; (c) suture over some form of temporary support, which will afterwards be dissolved by the action of the intestinal juices, and in relation to this he described the decalcified bone bobbin which he is in the habit of using. Among the disadvantages of the first method were the longer time which it required if interrupted sutures were employed, and the difficulty of preventing narrowing of the lumen of the canal if an attempt were made to use the continuous suture. As to Murphy's button, cases were related in which it had either gone astray or had been retained, and it was pointed out that, if any error were made in applying it, it might be impossible to unfasten it for the sake of purpose of readjustment, and that it had happened that, to set the bowel free, an operator had found it necessary to excise afresh the portion grasped by the button. The advantages of Mr. Mayo Robson's decalcified bone bobbins were that it was possible with their aid to bring the mucous membrane into accurate apposition, so as to protect the deeper layers and thus to avoid the production of that circle of granulation tissue which was the cause of after contraction; that by the use of the continuous suture, the two ends of which could be tied firmly together, the line of junction was held so firmly against the bobbin as to be rendered immediately water-tight, independently of the closeness of the stitches; that by the firmness with which the peritoneal suture, also a continuous one, could be tied, the silk was practically buried; that the whole process could be rapidly performed; and that, by virtue of the wide opening through the bobbin, the pent-up contents of the proximal portion of the bowel could immediately diffuse through the empty gut below, so soon as the clamps were removed. These points were demonstrated by Mr. Robson on a model after the meeting, and there seems little reason to doubt that these bobbins, made of decalcified bone, or potato, or turnip, or some such material, are likely to prove of very great service in abdominal surgery.

### Some Questions on Population.

During the decennium 1885-95, and for a long time previously, the population of France has been practically stationary. In that decennium she added but 67,100 to her numbers, whilst Great Britain increased by 2,452,400, Germany by 4,522,600, Russia by 12,510,800, and other European countries in proportions more or less similar. The questions that may be asked are such as these: What is the cause? And what is likely to be the effect of this condition of things? As to the cause, it seems to be principally twofold, an intense self-regard on the part of parents, and a nervous dread of evil for their offspring; this latter being merely an expansion, in sentiment, of the parental self-regard. In other words, the French, as a people, do not face the possibilities of life in a bold and resolute spirit, but with a timid and apprehensive nervousness. They bring up their children in their own spirit. The question is, Can a world like ours be mastered and made to yield its highest possible advantages to a people who face it in that spirit? In our judgment it cannot. What, then, is likely to be the final result of such action on the part of the French people if it be continued long enough? It seems that there can be but one result. That very thing must happen to a nerveless nation which happens to a nerveless individual. For a time such an individual holds his own with difficulty. Then he finds himself passed by, beaten, overwhelmed, subdued, and finally extinguished by a hundred forces bolder and more resolute than himself. Is Britain tending a little in the same direction as France? We hope not. We trust we may not see the last flicker of the national candle in our day. The next census will probably throw some clear light upon this point.

### Militant Charity.

Charity is subject to the fluctuations of fashion in common with other constituents of social life. The ways of Dr. Barnardo and his great Albert Hall displays are not those of the humble friar who collected the alms from givers in the Middle Ages; and the latest development of all, perhaps, is the military character of the methods adopted by those who would ameliorate the race physically or morally. We have the Salvation Army and the Church Army, both powerful charitable organisations, bent on treating their followers to an amount of discipline which demands rather than asks that they should give and do for others. The Chief Rabbi enforced this view of discipline at a recent meeting of the Shadwell Hospital for Children. He said on such an occasion all present should regard themselves under military orders, and feel it their duty to "present arms" (alms)! In respect to the laity, presence at a charity dinner is tantamount to the admittance of the obligation to give. Submission to discipline such as this was most aptly illustrated at an entertainment given by the Hon. Artillery Company a few nights ago in aid of Guy's Hospital. A football match was followed by a smoking concert, at which six hundred were present. During an interval the doors of the concert hall were closed, sentries were posted, and a collection was made. The company submitted to this military proceeding with great good humour, and the result of this enforcement of discipline will, it is believed, permit the Hon. Artillery Company to maintain two beds at Guy's Hospital during the present year, as they have done for some time past. So it is brought about that the military command to "present arms" is alike honoured by civilian and soldier.

